# Understanding and Overcoming Resistance to Selective FGFR inhibitors Across *FGFR2*-Driven Malignancies

**DOI:** 10.1158/1078-0432.CCR-24-1834

**Published:** 2024-11-01

**Authors:** Francesco Facchinetti, Yohann Loriot, Floriane Brayé, Damien Vasseur, Rastislav Bahleda, Ludovic Bigot, Rémy Barbé, Catline Nobre, David Combarel, Stefan Michiels, Antoine Italiano, Cristina Smolenschi, Lambros Tselikas, Jean-Yves Scoazec, Santiago Ponce-Aix, Benjamin Besse, Fabrice André, Ken A. Olaussen, Antoine Hollebecque, Luc Friboulet

**Affiliations:** 1 Université Paris-Saclay, Gustave Roussy, Inserm U981, Villejuif, France; 2 Département d'Innovation Thérapeutique (DITEP), Gustave Roussy, Villejuif, France; 3 Département de Médecine Oncologique, Gustave Roussy, Villejuif, France; 4 Medical Biology and Pathology Department, Gustave Roussy, Villejuif, France; 5 AMMICa UAR3655/US23, Gustave Roussy, Villejuif, France; 6 Département de Radiologie, Gustave Roussy, Villejuif, France; 7 Service de Pharmacocinétique, Faculté de Pharmacie, Université Paris-Saclay, Orsay, France; 8 Département de Biologie et Pathologies Médicales, Service de Pharmacologie, Gustave Roussy; 9 Université Paris-Saclay, Inserm, CESP, Villejuif, France; 10 Gustave Roussy, Office of Biostatistics and Epidemiology, Villejuif, France; 11 Faculty of Medicine, University of Bordeaux, Bordeaux, France; 12 BIOTHERIS, Department of Interventional Radiology, Gustave Roussy, Université Paris-Saclay, Villejuif, France; 13 Département de Biologie et Pathologie Médicales, Service de Pathologie Moléculaire, Gustave Roussy; AMMICa, CNRS UAR3655 INSERM US23; Université Paris-Saclay, Villejuif, France

**Keywords:** Cholangiocarcinoma, FGFR, resistance mechanisms, mutations, futibatinib, lirafugratinib

## Abstract

**Purpose:**

Understanding resistance to selective FGFR inhibitors is crucial to improve the clinical outcomes of patients with *FGFR2*-driven malignancies.

**Experimental Design:**

We analyzed sequential ctDNA, +/- WES or targeted NGS on tissue biopsies from patients with tumors harboring activating *FGFR2* alterations progressing on pan-FGFR-selective inhibitors, collected in the prospective UNLOCK program. FGFR2::BICC1 Ba/F3 and patient-derived xenografts (PDX) models were used for functional studies.

**Results:**

Thirty-six patients were included. In cholangiocarcinoma, at resistance to both reversible inhibitors (e.g. pemigatinib, erdafitinib) and the irreversible inhibitor futibatinib, polyclonal *FGFR2* kinase domain mutations were frequent (14/27 patients). Tumors other than cholangiocarcinoma shared the same mutated *FGFR2* residues, but polyclonality was rare (1/9 patients). At resistance to reversible inhibitors, 14 residues in the *FGFR2* kinase domain were mutated; after futibatinib, only the molecular brake N550 and the gatekeeper V565. Off-target alterations in PI3K/mTOR and MAPK pathways were found in 11 patients, often together with on-target mutations. At progression to a first FGFR inhibitor, 12 patients received futibatinib or lirafugratinib (irreversible inhibitors), with variable clinical outcomes depending on previous resistance mechanisms. Two patients with *TSC1* or *PIK3CA* mutations benefitted from everolimus. In cell viability assays on Ba/F3 and in pharmacologic studies on PDX, irreversible inhibitors retained better activity against *FGFR2* kinase domain mutations, with lirafugratinib active against the recalcitrant V565L/F/Y.

**Conclusions:**

At progression to FGFR inhibitors, *FGFR2*-driven malignancies are characterized by high intra- and inter-patient molecular heterogeneity, particularly in cholangiocarcinoma. Resistance to FGFR inhibitors can be overcome by sequential, molecularly-oriented treatment strategies across *FGFR2*-driven tumors.

## Introduction

Molecular alterations of fibroblast growth factor receptor family members (*FGFR1/2/3/4*) are frequent across cancers (1,2). *FGFR* amplifications are the most frequent alterations observed, yet their inconsistent oncogenic potential raises questions about their suitability as targets for selective inhibition (3). *FGFR2* gene fusions occur in 10-15% of intrahepatic cholangiocarcinoma cases, while activating mutations in the extracellular domain account for only a minor fraction of this malignancy (4–6). Recent research has highlighted the importance of deletions in the extracellular domain and truncations in the intracellular C-terminal domain of *FGFR2* as key drivers and therapeutic targets in intrahepatic cholangiocarcinoma (6,7). Of note, the same molecular alterations can be found, with a lower incidence, across a variety of solid tumors, of almost any histology (7).

The development and availability of selective FGFR inhibitors for treating *FGFR2*-driven intrahepatic cholangiocarcinoma are transforming the therapeutic landscape for patients with this molecular subtype (8–10). Selective FGFR inhibitors can be categorized into reversible (e.g. infigratinib, pemigatinib, erdafitinib, derazantinib, zoligratinib) and irreversible (e.g. futibatinib, lirafugratinib), based on their binding to the tyrosine kinase domain. Erdafitinib has demonstrated effectiveness in inhibiting FGFR3 in urothelial cancer (11,12), and shows activity across various *FGFR2*-driven tumors, reflecting the concept of molecularly-driven, tumor-agnostic targeted therapy (13). Similarly, pemigatinib and the two irreversible FGFR inhibitors, initially developed for *FGFR2*-driven intrahepatic cholangiocarcinoma, have shown efficacy across multiple tumor types in dose-expansion cohorts of phase I/II clinical trials (14–17).

Studies have identified frequent on-target, polyclonal mutations in the tyrosine kinase domain of *FGFR2* as common mechanisms of resistance to selective FGFR inhibitors in *FGFR2*-driven cholangiocarcinoma (18,19). These findings are supported by functional validation of specific *FGFR2* kinase domain mutations that confer resistance, with irreversible FGFR inhibitors designed to be effective against these mutations (20–26). Recently, off-target resistance mechanisms have been explored; pathogenic variants in the MAPK and PI3K/mTOR pathways have been detected at progression on selective FGFR inhibitors in patients with cholangiocarcinoma (21,23,27).

Importantly, resistance to FGFR inhibitors in the setting of *FGFR2*-driven disease has been predominantly reported in cholangiocarcinoma, except in four cases of *FGFR2*-driven tumors that progressed on pemigatinib (15,28). The broader application of FGFR inhibitors across different histologies underscores the need to identify and address resistance mechanisms in non-cholangiocarcinoma tumors, potentially offering universal strategies to counteract resistance.

In this study, we report on- and off-target resistance mechanisms to reversible and irreversible FGFR inhibitors across *FGFR2*-driven tumor types, validated through functional studies. Furthermore, the outcomes from sequential molecular treatments, applied to one-third of the patients with longitudinal monitoring of molecular alterations, provide insights into strategies to overcome resistance across a spectrum of *FGFR2*-driven solid tumors.

## Methods

### Patients and treatments

#### UNLOCK is an institutional program which aims to decipher mechanisms of action and resistance to innovative drugs

To be included in this cohort of the UNLOCK program, patients had to satisfy the following criteria: 1) Diagnosis of an advanced solid tumor requiring systemic treatment; 2) Molecular detection of an activating alteration (*i.e*. fusions/rearrangements, mutations) in the *FGFR2* gene; 3) Having received a selective FGFR inhibitor, either reversible (pemigatinib, erdafitinib, infigratinib, derazantinib, zoligratinib, rogaratinib, fexagratinib) or irreversible (futibatinib or lirafugratinib); 4) having post-progression molecular analyses performed on circulating tumor DNA (ctDNA) and/or tissue biopsies. In two cases showing primary resistance to futibatinib (***ST4455*** and ***MR719***), in the lack of the availability of post-progression samples, pre-treatment tissue biopsy and ctDNA were analyzed.

The molecular analyses were performed within four institutional studies at Gustave Roussy, whose aim is the molecular characterization of tumors: MATCH-R (NCT02517892) (29), MOSCATO (NCT01566019)(30), STING (NCT04932525), and CTC (NCT02666612).

Patients were treated in the setting of clinical trials or compassionate use programs allowing treatment with FGFR inhibitors on the basis of molecular selection. Disease response was measured according to RECIST 1.1, and progression-free survival (PFS) was calculated from the date of targeted inhibitor start to the day of radiological evidence of progression.

All patients participating in the mentioned studies were fully informed and signed a written informed consent. The studies have been approved by ethics committees in France (French National Agency for Medicines and Health Products Safety - ANSM), and are being conducted in accordance with the Declaration of Helsinki.

### Molecular analyses

Post-progression tissue biopsies, when possible, underwent whole exome sequencing (WES), with or without concomitant RNA sequencing (RNA-seq). The lower limit for WES performance was a proportion of tumor cells ≥ 30% in the tissue sample. In cases with a proportion of tumor cells between 10% and 30%, molecular analyses with targeted next-generation sequencing (NGS) panels (Mosc-4, Oncomine v3) were performed. For WES, the mean coverage was 140X.

With regard to ctDNA analyses, they were performed with GuardantHealth, Illumina, Foundation Medicine or Integragen liquid biopsy panels. For each patient with longitudinal ctDNA assessment, only analyses performed with the same platform were reported.

Among the findings of the molecular reports, only molecular events potentially implicated in resistance were reported in the present study. *FGFR2* kinase domain mutations were reported according to reference transcript NM_001144913.1, as previously reported by our group and others (20,21,31,32).

### Site-directed mutagenesis

Lentiviral vectors expressing *FGFR2:BICC1* fusions were created using the pLenti6/V5 directional TOPO Cloning Kit (#K495510, Thermo Fisher Scientific) according to the manufacturer's instructions. Point mutations in the *FGFR2* kinase domain of the *FGFR2::BICC1* fusion were introduced using the QuickChange XL Site-Directed Mutagenesis Kit (#200516, Agilent) according to manufacturer's protocol.

### Cell lines

Ba/F3 cells were infected with lentiviral constructs, as reported previously (33), to express the *FGFR2::BICC1* fusion, this latter with or without *FGFR2* kinase domain mutations. Ba/F3 cells harboring the fusion were selected in the presence of blasticidin (14 mg/mL) and IL-3 (0.5 ng/mL) until recovery, and a second selection by culturing the cells in the absence of IL-3. *FGFR2* fusion and *FGFR2* kinase domain mutations were confirmed on the established cell lines by Sanger sequencing. The cells were not tested for Mycoplasma contamination, but cells were not maintained in culture for more than two months after establishment or thawing.

Cell viability assays were performed in 96-well plates using the CellTiter Glo Luminescent Cell Viability Assay (G7570, Promega). We seeded 4000 cells/well and we treated cells for 48h. Half-maximal inhibitory concentration (IC_50_) values were derived using GraphPad Prism software.

### Reagents

Lirafugratinib was provided by Relay Therapeutics. Erdafitinib, infigratinib, fexagratinib, zoligratinib, derazantinib and futibatinib were purchased from Selleck Chemicals. Pemigatinib and rogaratinib were purchased from MedChemExpress.

### Development of patient-derived xenografts and *in vivo* pharmacologic studies

All animal procedures and studies have been approved by the French Ministry of “*Enseignement supérieur, de la Recherche et de l’Innovation*” (APAFIS#2790-2015112015055793 and APAFIS#2328-2015101914074846). Fresh tumor fragments were implanted in the subrenal capsule of 6-week-old female NOD/SCID gamma (NSG) mice obtained from Charles River Laboratories. Patient-derived xenografts (PDX)-bearing NSG mice were treated with the indicated doses of pemigatinib, erdafitinib, futibatinib and lirafugratinib. Eight mice per group were treated for up to 50 days, and tumor volume and mouse weight were measured twice weekly.

## Results

### Patient population and molecular treatments

We studied 36 patients with advanced solid tumors driven by *FGFR2*, all of whom were progressing on selective FGFR inhibitors (Supplementary Table S1). This cohort included 27 patients with intrahepatic cholangiocarcinoma and nine patients with various other tumor types: two with high-grade serous ovarian cancer, and one each with lung adenocarcinoma, urothelial cancer, triple-negative breast cancer, duodenal cancer, pancreatic ductal adenocarcinoma, adrenocortical carcinoma, and cancer of unknown primary. The majority, 31 patients, had tumors harboring *FGFR2* fusions, while five had tumors driven by *FGFR2* mutations located in the extracellular domain (specifically, three with FGFR2 C383R, one with FGFR2 S267P, and one with FGFR2 Y376C). *FGFR2* fusion partners included *BICC1* in five cases (all in intrahepatic cholangiocarcinomas), *TACC2* in three cases (n = 1 intrahepatic cholangiocarcinoma, n = 2 other tumor types), *STRN4* and *CCSSER2* in two cases, one from each cohort. Other unique fusion partners were found in the remaining 19 tumors, detailed in Supplementary Tables S2-3.

Twenty-three patients received a reversible FGFR inhibitor (n = 13 pemigatinib, n = 8 erdafitinib, n = 1 derazantinib, n = 1 zoligratinib), and 13 the irreversible inhibitor futibatinib (Supplementary Table S1). In the cholangiocarcinoma group, patients treated with reversible inhibitors and futibatinib showed 61% and 67% objective response rate, with median PFS of 8.7 and 11.1 months, respectively (Supplementary Table S4). Given the diversity in tumor origin, detailed clinical data of the non-cholangiocarcinoma patients is provided in Supplementary Table S3.

All patients underwent post-progression ctDNA analysis. Twenty-one of them had further molecular analyses performed on post-progression tissue biopsies, 16 via whole exome sequencing (WES) with or without RNA sequencing (RNA-seq), and five via targeted next-generation sequencing (NGS).

After disease progression on the first FGFR inhibitor, 12 patients received sequential targeted treatments. Eight patients with cholangiocarcinoma received futibatinib following a reversible inhibitor, with three receiving the mTOR inhibitor everolimus based on molecular findings. Two patients with cholangiocarcinoma and two with other tumor types, progressing respectively on pemigatinib and futibatinib, were switched to the FGFR2-selective inhibitor lirafugratinib (Supplementary Table S1).

### Molecular alterations observed at resistance to selective FGFR inhibitors

In order to better approach the specificities of resistance mechanisms to reversible inhibitors *versus* the irreversible inhibitor futibatinib, and between intrahepatic cholangiocarcinoma and other tumor types, we separated below the different groups of patients analyzed at progression after a first FGFR inhibitor.

#### Intrahepatic cholangiocarcinoma on reversible inhibitors

**Figure 1:**
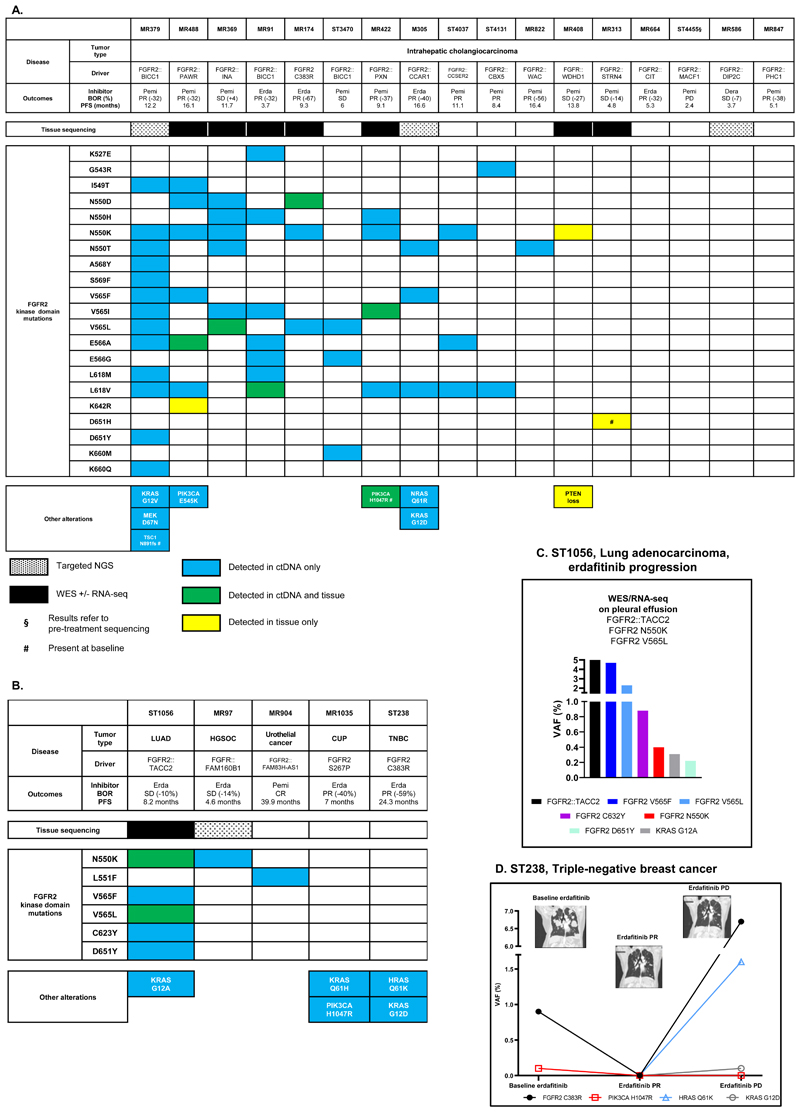
Molecular findings at resistance to reversible FGFR inhibitors. **A:** Patients suffering from intrahepatic cholangiocarcinoma. **B:** Patients suffering from other tumor types. **C:** Molecular findings of patient ***ST1056***, suffering from a lung adenocarcinoma harboring a *FGFR2::TACC2* fusion, at acquired progression to erdafitinib. **D:** Clinico-radiological and molecular evolution of patient ***ST238***, suffering from a FGFR2 C383R-driven triple-negative breast cancer. The ctDNA findings are reported as variant allele frequency (VAF, %). BOR: Best objective response; PFS: Progression-free survival; PR: Partial response: SD: Stable disease; PD: Progressive disease; Pemi: Pemigatinib; Erda: Erdafitinib; Dera: Derazantinib; LUAD: Lung adenocarcinoma; HGSOC: High-grade serous ovarian cancer; CUP: Cancer of unknown primary; TBNC: Triple-negative breast cancer; CR: Complete response.

The molecular alterations detected in 17 patients with intrahepatic cholangiocarcinoma progressing on reversible inhibitors are reported in Figure 1A. Polyclonal kinase domain mutations (≥ 2 *FGFR2* mutations in the same blood sample) were detected in 10 of these patients (59%).

In three additional patients, a single *FGFR2* mutation was detected either in the tissue biopsy (***MR408*** and ***MR313***) or in ctDNA ***(MR822***; FGFR2 N550T). Specifically, FGFR2 D651H was detected in both pre- and post-treatment biopsies of one patient (***MR313***). Another unique case (***MR488***) had two concurrent *FGFR2* kinase domain mutations in a single tissue biopsy (E566A and K642R). Concurrent pathogenic alterations in the MAPK and PI3K/mTOR pathways, suggesting off-target resistance mechanisms, were observed in five patients.

#### Other tumor types on reversible inhibitors

Five patients with non-cholangiocarcinoma *FGFR2*-driven tumors exhibited diverse resistance patterns after initial tumor shrinkage (Figure 1B).

*FGFR2* kinase domain mutations were detected in three of these patients, with one exhibiting polyclonal mutation. The latter (patient ***ST1056)***, was a *FGFR2::TACC2* rearranged lung adenocarcinoma that progressed on erdafitinib with FGFR2 N550K, V565L/F, C632Y, D651Y mutations, as well as KRAS G12A (Figure 1C, Supplementary Figure S1).

In two patients, no *FGFR2* kinase domain mutations aroused at progression to erdafitinib (***MR1035***, cancer of unknown primary; ***ST238***, triple negative breast cancer, Figure 1D), but *KRAS*/*PIK3CA* and *HRAS*/*KRAS* mutations were detected in ctDNA at progression, respectively (Figure 1B).

#### Intrahepatic cholangiocarcinoma on futibatinib

**Figure 2:**
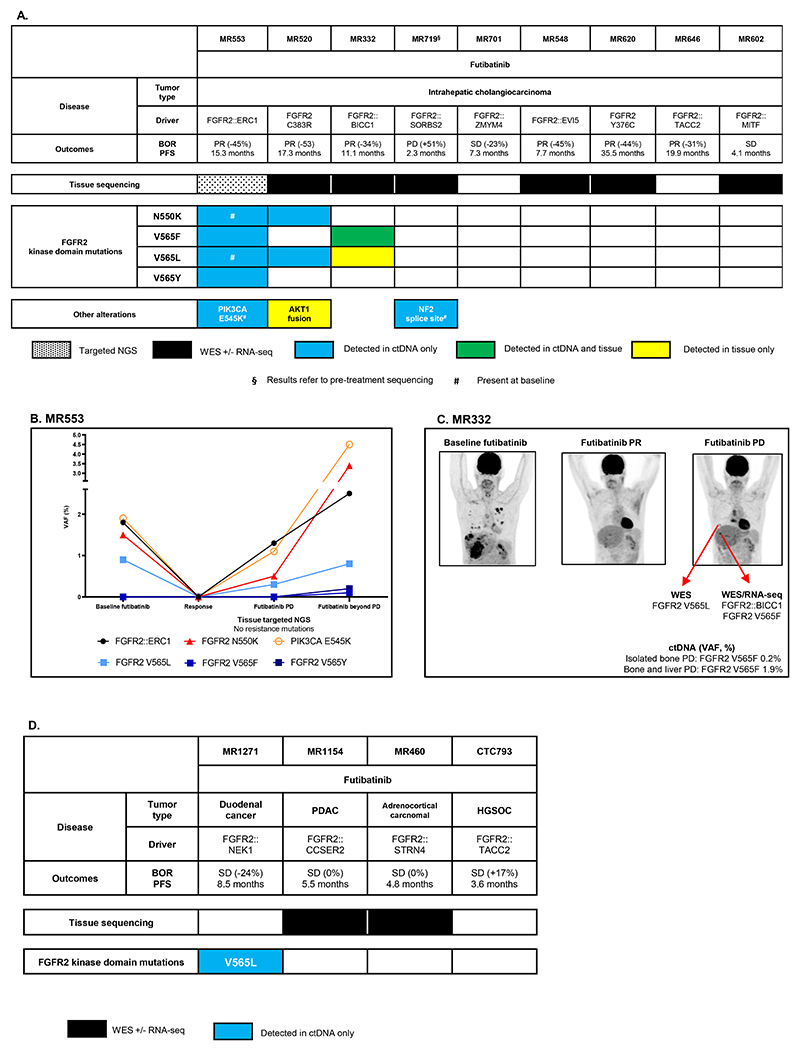
Molecular findings at resistance to the irreversible FGFR inhibitor futibatinib. **A:** Patients suffering from intrahepatic cholangiocarcinoma. **B:** Molecular evolution of patient ***MR553***, suffering from an intrahepatic cholangiocarcinoma harboring a *FGFR2::ERC1* fusion. **C:** Clinico-radiological and molecular evolution of patient ***MR332***, suffering from an intrahepatic cholangiocarcinoma driven by a *FGFR2::BICC1* fusion. **D:** Patients suffering from other tumor types. The ctDNA findings are reported as variant allele frequency (VAF, %). BOR: Best objective response; PFS: Progression-free survival; PR: Partial response: SD: Stable disease; PD: Progressive disease; PDAC: Pancreatic ductal adenocarcinoma; HGSOC: High-grade serous ovarian cancer.

Among nine patients with intrahepatic cholangiocarcinoma progressing on futibatinib, fewer *FGFR2* kinase domain mutations were observed compared to those on reversible inhibitors, mainly involving key regions like the molecular brake (N550) and gatekeeper (V565) (Figure 2A). Only three patients had polyclonal mutations, which were limited to N550K and V565F/L/Y.

One patient (***MR553***) showed pre-treatment *FGFR2* kinase domain mutations that disappeared during response and re-emerged at progression (Figure 2B, Supplementary Figure S2).

Interestingly, the patient ***MR332*** firstly experienced an isolated bone progression, whose biopsy revealed a *FGFR2* V565L mutation (not detectable in blood), followed by a hepatic progression harboring a *FGFR2* V565F mutation (Figure 2C).

#### Other tumor types on futibatinib

**Figure 5:**
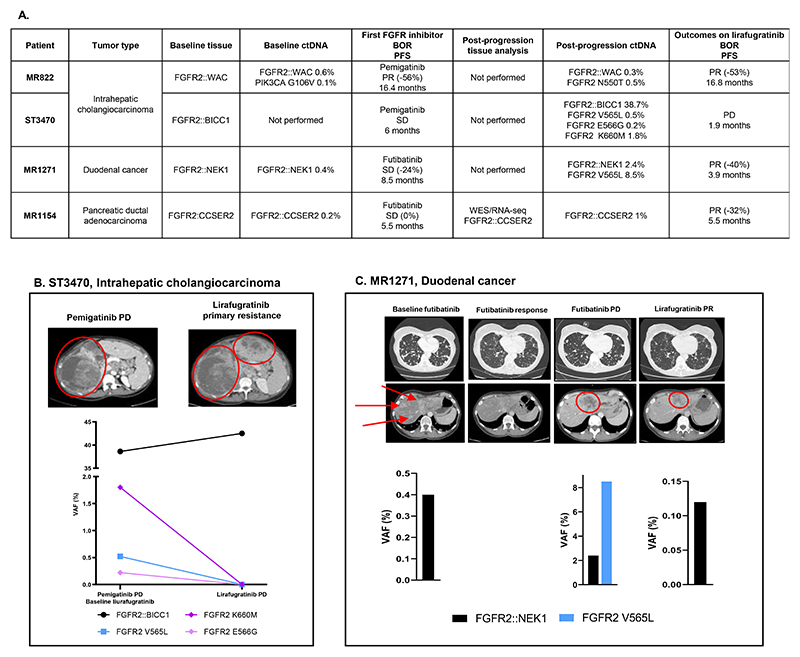
Clinical and molecular evolution of patients receiving a first FGFR inhibitor followed by the irreversible, highly-FGFR2 selective inhibitor lirafugratinib. **A:** Four patients harboring a *FGFR2* fusion received lirafugratinib as a second FGFR inhibitor. None received intercurrent treatment between the FGFR inhibitors. **B:** Clinico-radiological and molecular evolution of patient ***ST3470***, with a cholangiorcarcinoma driven by *FGFR2::BICC1* progressing on pemigatinib. **C:** Patient ***MR1271*** had duodenal cancer progressing on futibatinib with emergence of FGFR2 V565L mutation, that was cleared by lirafugratinib. The ctDNA findings are reported as variant allele frequency (VAF, %). BOR: Best objective response; PFS: Progression-free survival; PR: Partial response; SD: Stable disease; PD: Progressive disease.

Four patients with various tumor types showed resistance mechanisms to futibatinib (Figure 2D), including a duodenal cancer patient (***MR1271***) who exhibited a monoclonal FGFR2 V565L mutation concurrent with progression in the lung and liver (see Figure 5C).

### Global analysis of candidate resistance mechanisms

**Figure 3:**
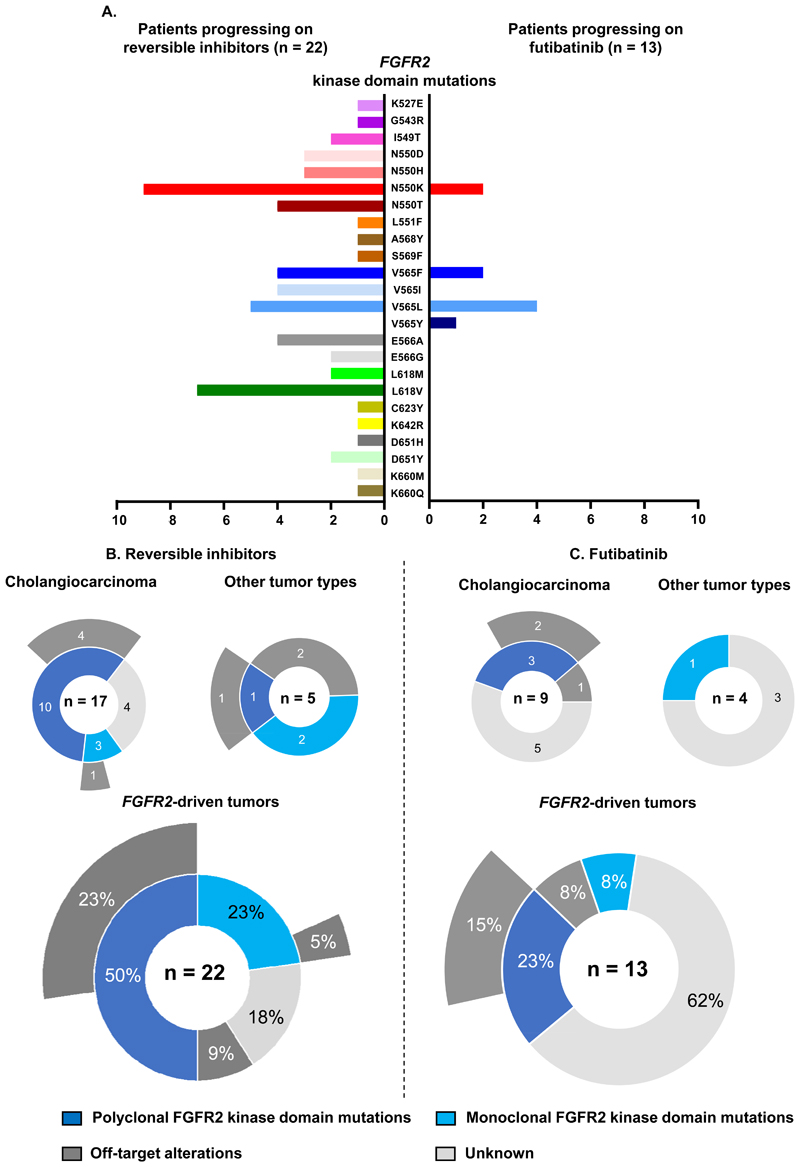
Global view on candidate resistance mechanisms to FGFR inhibitors across *FGFR2*-driven malignancies. **A:** Spectrum of FGFR2 kinase domain mutations detected across patients progressing to reversible inhibitors and futibatinib. **B:** Overview of the molecular alterations found at progression to reversible inhibitors in cholangiocarcinoma and other tumor types (upper panels), and pooled across all cases. **C:** Overview of the molecular alterations found at progression to the irreversible inhibitor futibatinib in cholangiocarcinoma and other tumor types (upper panels), and pooled across all cases.

Comparing the spectrum of putative resistance mechanisms occurring in *FGFR2-*driven cholangiocarcinoma or other tumor types, we hypothesized that the two entities converged towards overlapping ways to escape targeted FGFR2 inhibition. We therefore pooled the molecular data of the two populations to allow a global view on resistance to a first FGFR inhibitor among *FGFR2*-driven tumor types (Figure 3).

Across the 36 patients, 14 residues in the FGFR2 kinase domain (K527, G543, I549, N550, L551, A568, S569, V565, E566, L618, C623, K642, D651, K660) were found mutated at progression. For six of them (N550, V565, E566, L618, D651 and K660), at least two possible substitutions were observed, thus representing 24 possible mutations (Figure 3A). FGFR2 C623Y and L551F were the only mutations found exclusively in non-cholangiocarcinoma (Figure 1B). FGFR2 C623Y has not previously been reported, while L551F has been described in the setting of cholangiocarcinoma progressing on infigratinib (19).

Polyclonal kinase domain mutations were detected in half (11/22) of the patients progressing on reversible inhibitors, almost exclusively with cholangiocarcinoma, whereas only three patients (23%) revealed polyclonal *FGFR2* mutations after futibatinib (Figure 3B-C). When assessable due to amplicon sizes, the polyclonal *FGFR2* kinase domain mutations were always detected *in trans* (*i.e*. on different alleles).

Overall, 61 *FGFR2* kinase domain mutations were detected after reversible inhibitors, while only nine mutations were observed after futibatinib (Figure 3A). The most frequently mutated residues were the molecular brake N550 and the gatekeeper V565.

In 13/22 (59%) of the patients progressing on reversible inhibitors, at least one mutation affecting either of these two residues was found, whereas the two residues were co-mutated in eight cases (36%). N550 and V565 were also the residues with the highest number of different substitutions, as N550 D/H/K/T and V565F/I/L. L618V/M occurred in nine cases, followed by E566 (E566A/G) that was mutated in six patients.

In contrast, the N550 and V565 residues were the unique sites of *FGFR2* kinase domain mutations found at progression to futibatinib, namely N550K (n = 2), V565F (n = 2), V565L (n = 4), and V565Y (n = 1) (Figure 2A, 3A). Of note, the mentioned molecular brake and gatekeeper mutations have been previously reported, with the exception of FGFR2 V565Y (34). This mutation is a novel entity, emerging from a double-base substitution in the corresponding Valine codon GTT, for which we hypothesize the sequential occurrence of single nucleotide substitutions, from V565F (F being coded by the codon TTT) to V565Y (Y being coded by TAT).

Off-target mutations that are potentially implicated in resistance, such as those affecting the MAPK (*i.e. HRAS, KRAS, NRAS, MEK*) and PI3K/mTOR pathways (*i.e. PIK3CA, PTEN, TSC1*), were found in 8/22 (36%) and 3/13 (23%) cases progressing on reversible inhibitors and futibatinib, respectively (Figure 1A-B, 2A, 3B-C). In eight patients (23%), these mutations co-occurred with *FGFR2* kinase domain mutations. In two patients with other tumor types progressing on erdafitinib, they emerged without concomitant on-target alterations (Figure 1B).

Pooling the molecular data obtained at progression, we observed molecular candidates for resistance in 77% and 38% of the patients progressing on reversible inhibitors and futibatinib, respectively (Figure 3B-C).

### Sequential treatment strategies

Within our study cohort, 33% of the patients (n = 12, including 10 with intrahepatic cholangiocarcinoma and one each from pancreatic and duodenal cancers) underwent sequential targeted therapy regimens (with longitudinal sampling) that included irreversible FGFR inhibitors and the mTOR inhibitor everolimus.

#### Sequential treatments including futibatinib and everolimus

**Figure 4:**
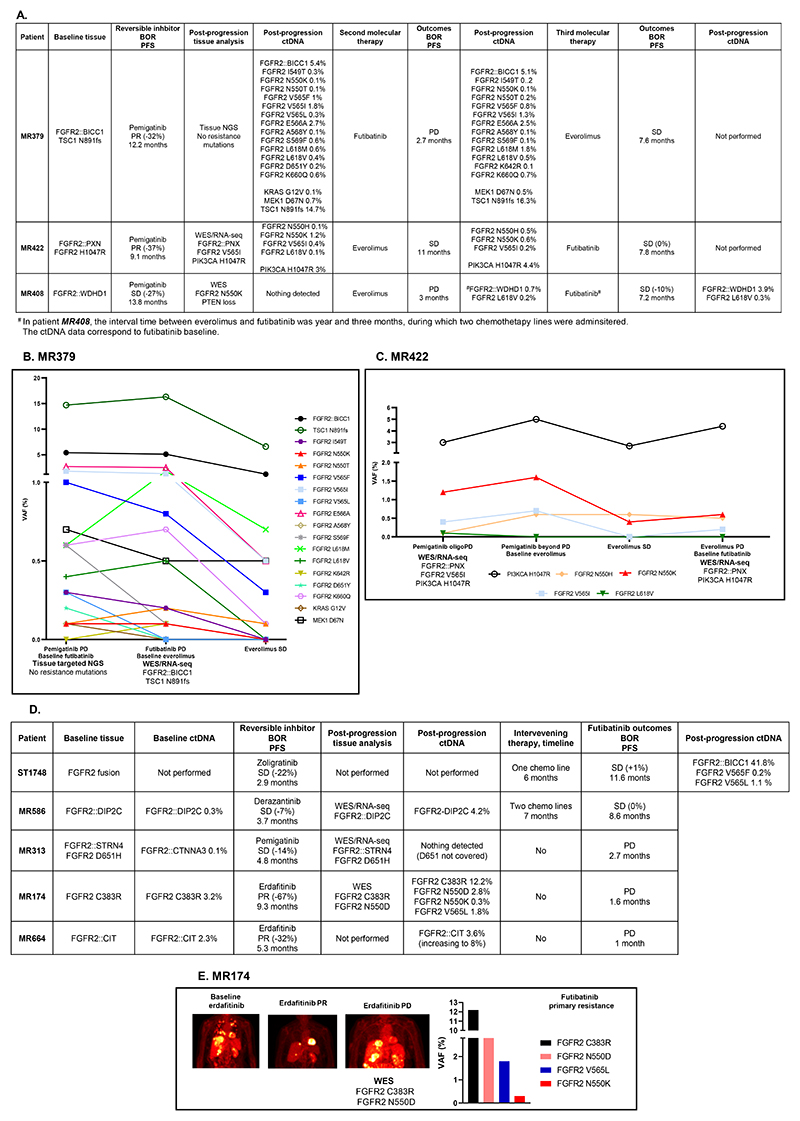
Clinical and molecular evolution of patients with *FGFR2*-driven intrahepatic cholangiocarcinoma receiving sequential targeted treatments including futibatinib and everolimus. **A:** Three patients received a sequential treatment of futibatinib and everolimus, this latter administered given the molecular finding of alterations in the PI3K/mTOR pathway. **B:** Molecular evolution of patient ***MR379***, suffering from an *FGFR2::BICC1* driven disease, with a concomitant pathogenic *TSC1* frameshift mutation. **C:** Molecular evolution of patient ***MR422***, suffering from a *FGFR2*-rearranged disease, with a concomitant PIK3CA H1047R mutation. **D:** Additional five patients received a sequence of reversible FGFR inhibitor followed by futibatinib. **E:** Clinico-radiological and molecular evolution of patient ***MR174***, suffering from a FGFR2 C383R driven disease. The ctDNA findings are reported as variant allele frequency (VAF, %). BOR: Best objective response; PFS: Progression-free survival; PR: Partial response; SD: Stable disease; PD: Progressive disease; Chemo: Chemotherapy.

After progressing on reversible inhibitors, three patients with intrahepatic cholangiocarcinoma received a sequential treatment with futibatinib and everolimus based on specific molecular findings (Figure 4A):

Patient ***MR379*** suffered from a tumor harboring both *FGFR2::BICC1* fusion and a *TSC1* frameshift mutation in the baseline tissue sample. At progression to pemigatinib, the two alterations were found in ctDNA together with 13 different *FGFR2* kinase domain mutations, *KRAS* and *MEK1* mutations (Figure 4B). Futibatinib did not induce any clinical benefit (stable disease; PFS 2.7 months), while everolimus, administered due to the *TCS1* loss-of-function alteration, led to stable disease with a PFS of 7.6 months. The clinical benefit was accompanied by the reduction of the allele frequencies of all the alterations in ctDNA (Figure 4B).

Patient ***MR422*** experienced oligo-progression while on pemigatinib. A liver biopsy revealed a new FGFR2 V565I mutation alongside pre-existing *FGFR2::PNX* fusion and PIK3CA H1047R mutation (Figure 4A, C). Despite continued progression, pemigatinib treatment was extended, resulting in increased variant allele frequency (VAF) of PIK3CA H1047R, FGFR2 V565I, N550H and N550K mutations. Everolimus, initiated due to the *PIK3CA* mutation, provided stable disease for 11 months, corresponding to a decrease in the VAF of the documented mutations. Their VAF increased again at everolimus progression, and subsequent futibatinib administration allowed the achievement of disease stabilization with a PFS of 7.8 months.

Patient ***MR408*** progressed to pemigatinib with an isolated lung nodule showing a FGFR2 N550K mutation and *PTEN* loss. Although everolimus showed no clinical activity, subsequent futibatinib treatment led to tumor shrinkage and a PFS of 7.2 months, despite the baseline documentation of a FGFR2 L618V mutation (Supplementary Figure S3).

#### Sequential treatments with reversible FGFR inhibitors followed by futibatinib

Five additional patients with *FGFR2*-driven cholangiocarcinoma were treated with futibatinib after experiencing resistance to reversible FGFR inhibitors (Figure 4D).

Two patients showed clinical benefit from futibatinib (***MR586*** and ***ST1748***). Upon progression on futibatinib, FGFR2 V565F/L mutations were identified in patient ***ST1748***, consistent with mutations typically seen in FGFR inhibitor-naïve patients. Three other patients experienced primary resistance to futibatinib after acquired resistance to reversible inhibitors. Of notice, FGFR2 N550D/K and V565L were present at futibatinib baseline in ***MR174***, and could explain its lack of benefit (Figure 4E).

#### Sequential treatments with lirafugratinib

Four patients received lirafugratinib after progressing on a previous inhibitor (pemigatinib or futibatinib), without other intervening therapies (Figure 5A).

Lirafugratinib outcomes were divergent among the two patients with *FGFR2*-rearranged cholangiocarcinoma progressing on pemigatinib.

Patient ***MR822***, with a prolonged initial response to pemigatinib, developed an FGFR2 N550T mutation along with a persistent driver fusion *FGFR2::WAC*. Lirafugratinib treatment resulted in another prolonged response, highlighting its effectiveness against this specific mutation (Supplementary Figure S4A). Patient **ST3470** encountered primary progression on lirafugratinib despite no detectable *FGFR2* kinase domain mutations in ctDNA, suggesting an alternative resistance mechanism (Figure 5B). Importantly, three FGFR2 mutations V565L, E566G, and K660M present before lirafugratinib were lost at progression, suggesting their sensitivity to lirafugratinib.

Two other patients suffering from tumors other than cholangiocarcinoma also benefited from lirafugratinib after futibatinib progression (Figure 5A).

Patient ***MR1271*** suffered from a *FGFR2::NEK1* driven duodenal carcinoma, who progressed on futibatinib with the acquisition of FGFR2 V565L in ctDNA, and major disease response was observed with lirafugratinib (Figure 5C).

Patient ***MR1154*** suffered from a pancreatic carcinoma harboring *FGFR2::CCSER2* fusion. No molecular events potentially implicated in resistance to futibatinib were detected, but *FGFR2::CCSER2* VAF was no longer detectable three weeks after lirafugratinib initiation (Supplementary Figure S4B).

### *FGFR2* kinase domain mutations exert a differential spectrum of resistance according to selective FGFR inhibitors

**Figure 6:**
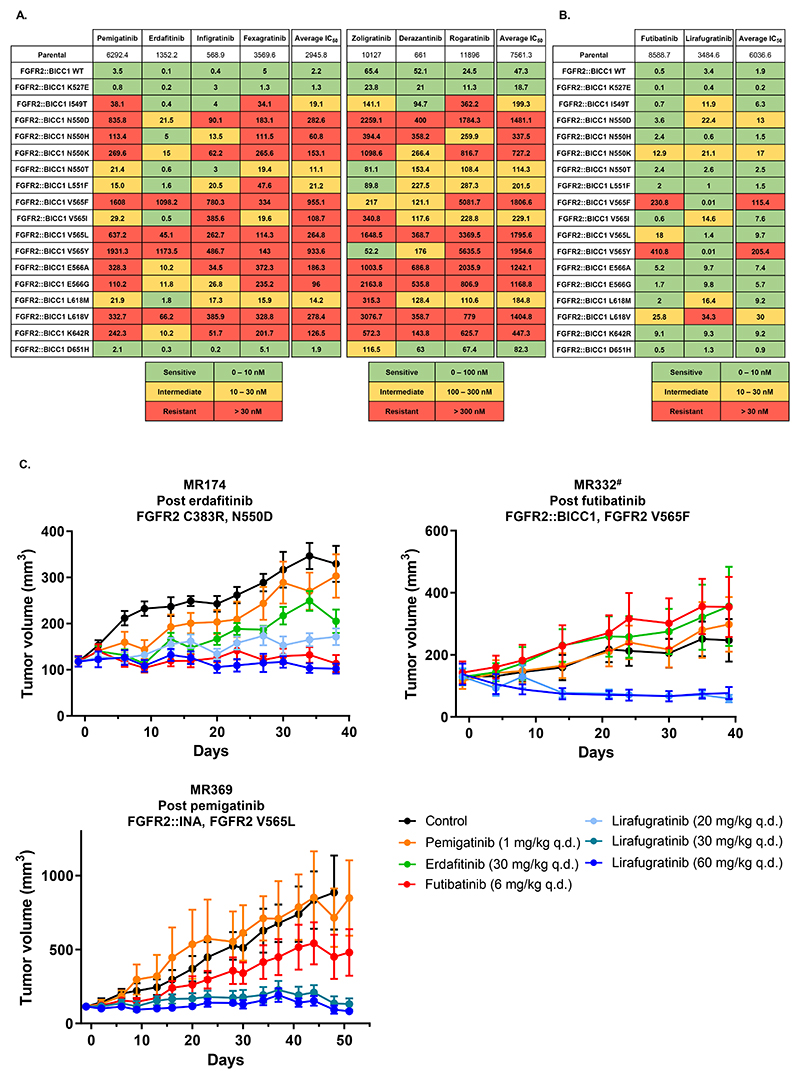
*In vitro* and *in vivo* evaluation of the activity of selective FGFR inhibitors against FGFR2 kinase domain mutations. **A:** IC_50_ values of seven reversible FGFR inhibitors (and their average) against parental Ba/F3, FGFR2::BICC1 Ba/F3 (wild-type, WT), and 17 mutants. **B:** Graphical representation of the IC50 values of the two irreversible FGFR inhibitors futibatinib and lirafugratinib (and their average) against parental Ba/F3, FGFR2::BICC1 Ba/F3 (WT), and 17 mutants. We created two different cut-off thresholds, given the lower potency of zoligratinib, derazantinib and rogaratinib against WT FGFR2::BICC1 Ba/F3. In **A** and **B**, IC_50_ values (nmol/L) are reported as means of ≥ 3 independent datasets. **C:** Tumor growth kinetics in PDX models established from patients with cholangiocarcinoma, exposed to four FGFR inhibitors. ^#^ ***MR332*** PDX was established from the liver tissue biopsy harboring FGFR2 V565F, while the bone lesion harbored FGFR2 V565L (see Figure 2C). q.d.: *quaque die* (*i.e*. daily).

To explore how specific *FGFR2* kinase domain mutations affect resistance to FGFR inhibitors, we used 18 Ba/F3 cell lines, engineered to express the *FGFR2::BICC1* fusion with various secondary mutations. *FGFR2::BICC1* was chosen being the most frequent fusion observed in our cohort and in other series (35). We exposed each Ba/F3 cell line to increasing concentrations of seven selective, reversible FGFR inhibitors and to the irreversible agents futibatinib and lirafugratinib, in order to establish their IC_50_ (Figures 6A-B, Supplementary Figure S5).

In our experiments, erdafitinib emerged as the most potent inhibitor across all mutants, followed closely by infigratinib and futibatinib, achieving sub-nanomolar IC_50_ values against the wild-type FGFR2::BICC1 Ba/F3 cell line (Figure 6A-B).

The profiles of sensitivity and resistance conferred by individual *FGFR2* kinase domain mutations matched with the spectrum of mutations emerging in patients treated with either reversible agents or futibatinib, respectively (Figure 3A, 6A-B). FGFR2 D651H did not confer resistance to any of the inhibitors, suggesting its role as a passenger event in patient ***MR313***.

A significant finding from our study was the variable resistance patterns conferred by mutations at key residues within FGFR2, notably the molecular brake N550 and the gatekeeper V565. Mutations at these sites-N550K and V565F/L/Y-broadly conferred resistance across several reversible inhibitors, casting doubt on the effectiveness of using these drugs sequentially in patients with these mutations. However, other mutations like N550T and V565I seemed to result in a lesser degree of resistance. Interestingly, zoligratinib demonstrated relatively lower IC50 values against V565F/Y mutations compared to those against V565I/L, indicating specific interactions between the inhibitor and variant amino acids at this site.

The irreversible inhibitors generally showed superior activity compared to reversible agents in our Ba/F3 models. Both futibatinib and lirafugratinib exhibited efficacy within the 2-20 nmol/L range against various mutations, although they displayed slight differences in activity against certain mutations. Notably, lirafugratinib was particularly effective against FGFR2 V565F/Y mutations-mutations where futibatinib showed reduced activity due to steric hindrances from bulky amino acids like Phenylalanine and Tyrosine (IC_50_ > 200 nmol/L) (25,36). In contrast, the two irreversible inhibitors showed an opposite profile of activity against FGFR2 V565I and V565L mutants, with futibatinib being more active on V565I and lirafugratinib on V565L. The slightly difference in tridimensional structure between Leucine (V565L) and Isoleucine (V565I) likely explains the activity of the two inhibitors. In addition, FGFR2 V565I is known to increase basal activity of the kinase domain (37), and the higher potency of futibatinib could prevail in this setting.

The two irreversible inhibitors maintained an IC_50_ in the 10-20 nmol/L range for FGFR2 N550K, the most common mutation arising after a reversible inhibitor. Since FGFR2 N550K occurred in two patients progressing on futibatinib, and in five patients the gatekeeper FGFR2 V565L emerged (Figures 2A, 4D), we suppose that these two mutations cannot be overcome with clinically achievable concentrations of the agent.

In order to further validate our *in vitro* preclinical analyses on more clinically relevant models *in vivo*, we established PDXs from biopsies of patients with cholangiocarcinoma, collected at the time of acquired resistance to FGFR inhibitors. We treated three PDX models with pemigatinib, erdafitinib, futibatinib and lirafugratinib (Figure 6C). The FGFR2 N550D mutation in ***MR174*** PDX, established at progression to erdafitinib, could be overcome by futibatinib and lirafugratinib. In addition, a dose-effect was noticed for lirafugratinib, with tumor growth abrogated only at the dose of 60 mg/kg (and not 20 mg/kg), which is in line with the IC_50_ observed in the Ba/F3 models. ***MR369*** PDX, established at progression to pemigatinib, harbored a FGFR2 V565L mutation, while ***MR332*** PDX, established at futibatinib resistance, harbored FGFR2 V565F (see Figure 2C). In both cases, only lirafugratinib (even at low doses) was able to prevent tumor growth in this *in vivo* model, confirming our suggestion that gatekeeper mutations FGFR2 V565L/F can be difficult to overcome with futibatinib in the clinical setting, while retaining sensitivity to lirafugratinib.

Of note, two of the PDX models (***MR174*** and ***MR369***) were established from patients progressing with polyclonal *FGFR2* kinase domain mutations detected in ctDNA, but only one mutation was found in the corresponding tissue biopsy and PDX (Figure 6C). These observations underscore the limitation of tissue biopsies to fully recapitulate the molecular spectrum of heterogeneity observed in patients at resistance.

## Discussion

FGFR inhibition in *FGFR2*-driven malignancies marks a significant advance in precision oncology, emphasizing the need to understand molecular mechanisms behind drug resistance to develop new treatment strategies. Our study integrates extensive clinical and molecular data, along with *in vitro* and *in vivo* validation assays, to explore resistance mechanisms in patients with *FGFR2*-driven cancers, including cholangiocarcinoma and other tumor types.

Our findings confirm that polyclonality of *FGFR2* kinase domain mutations is commonly observed in ctDNA from patients at progression on reversible inhibitors, particularly in cholangiocarcinoma. (18–20). Indeed, *FGFR2* kinase domain mutations were undetectable or found as isolated entities in tissue analyses, compared to multiple alterations in ctDNA, highlighting the "polyclonal" nature of tumor progression and the fundamental role of liquid over tissue biopsy. Interestingly, such mutations were less common after treatment with the irreversible inhibitor futibatinib, which primarily affected the molecular brake N550 and gatekeeper V565 residues.

Recently, Wu and colleagues gathered data on resistance mechanisms in patients with *FGFR2*-driven cholangiocarcinoma, pooling evidence from published papers and meeting abstracts (21). In our study, we were able to differentiate between resistance to reversible inhibitors and futibatinib, providing clinical proof to their functional observations. As predicted by their evaluation of clinically achievable doses of futibatinib, in our cohort FGFR2 N550K frequently emerged at progression to the irreversible agent. Interestingly, we did not detected any mutation in the binding site for irreversible inhibitors (FGFR2 C492), in line with the reduced cellular fitness caused by these mutations, which somehow suggests their limited frequency of occurrence, such as the FGFR2 C492F found in the patient reported by Berchuck and colleagues (32). On the other hand, FGFR2 V565L, labeled by Wu and colleagues as sensitive to futibatinib, was the mutation most frequently observed at progression to the irreversible inhibitor in our cohort.

Similarly, resistance mechanisms in tumors other than cholangiocarcinoma mirrored those observed in cholangiocarcinoma, involving known *FGFR2* residues and off-target resistance mechanisms. If considering the nine patients with other tumor types in our cohort, together with the report from Nicolò and colleagues (FGFR2 V565L detected at pemigatinib progression in a patient with breast cancer) (28), the emergence of polyclonal *FGFR2* mutations was limited to only one patient with a lung adenocarcinoma progressing on erdafitinib (Figure 1C). More recently nevertheless, Rodón and colleagues detected polyclonal *FGFR2* mutations in two patients with non-cholangiocarcinoma tumors progressing on pemigatinib (15). Whatsoever, the overall small number of patients with *FGFR2*-driven other tumor types evaluated at resistance challenges the conclusion that the propensity of developing polyclonal *FGFR2* mutations is a feature more common in *FGFR2*-driven cholangiocarcinoma.

Further, in concomitance with *FGFR2* kinase domain mutations or not, alterations in genes of the PI3K/mTOR pathway were frequently present at progression to reversible inhibitors and futibatinib, in patients suffering from cholangiocarcinoma or from other tumor types. Interestingly, in three patients, clinical benefit was obtained from reversible FGFR inhibitors or futibatinib, despite the presence of PI3K/mTOR alterations at baseline (***MR379, MR422*** and ***MR553***), which were maintained at progression (Figure 1A and 2A). In line with our clinical observations, Wu and colleagues recently reported that the PI3KCA E545K mutation does not impact futibatinib sensitivity in the context of *FGFR2*-driven cholangiocarcinoma cell lines (21).

Here, the mTOR inhibitor everolimus provided clinical benefit in two patients with alterations in *TSC1* or *PIK3CA* at progression to FGFR inhibition. The reduction in VAF of concomitant *FGFR2* kinase domain mutations during everolimus treatment suggests that the on-target alterations probably emerged as an early event in a tumor clone already harboring the corresponding *TSC1* or *PI3KCA* mutations (Figure 4B-C). It therefore seems that the loss of function of *TSC1* and the activation of *PIK3CA* do not represent the *bona fide* molecular mechanisms responsible for resistance to FGFR inhibitors, still explaining the clinical benefit from everolimus.

The enrichment in MAPK pathway alterations in a setting similar to ours has been recently reported by DiPeri and colleagues in cholangiocarcinoma (27). Whether these mutations can be overcome by combination treatment in the clinical setting is still to be proven, as according to their data, only an *in vitro* synergistic effect of FGFR/MEK inhibition was achieved, with no meaningful effect in the *in vivo* model.

In the present study, irreversible inhibitors were also administered after progression to reversible ones in one third of the patients. We integrated the case-by-case analysis of the clinical response in presence of precise *FGFR2* kinase domain mutations, with the dynamics of resistance mutations in ctDNA during treatment sequencing, and exploring functional data in Ba/F3 cellular models and matched PDX models. Nevertheless, the unique complexity of *FGFR2*-driven tumors at progression to reversible inhibitors, in terms of high levels of molecular heterogeneity, hampers the definition of precise patterns of resistance suitable for the sequential treatment with an irreversible agent. This is in contrast with single on-target mutations in *EGFR*- and *ALK*- driven lung cancer, overcome by the respective third generation inhibitors in the clinical setting (38,39). In our cohort and in line with other reports (6,40), in case of progression to a first FGFR inhibitor (reversible or futibatinib) mediated by a unique *FGFR2* kinase domain mutation, the clinical activity profiles of futibatinib and lirafugratinib corresponded well to our functional assessment. We were indeed able to overcome resistance to reversible inhibitors and futibatinib due to mutations occurring in the *FGFR2* gatekeeper residue (FGFR2 V565F/L). In line with the initial proofs from Subbiah and colleagues (25), the FGFR2-selective inhibitor lirafugratinib was active in our Ba/F3 cellular models, PDX and in patient ***MR1271*** (FGFR2 V565L).

On the other hand, objective responses to futibatinib and lirafugratinib were observed even in cases with polyclonal *FGFR2* kinase mutations (20,25). The relative abundance of each individual resistance mutation at baseline of the irreversible inhibitors, is suspected to influence the clinical response on a systemic scale. Emblematic in this sense is the evolution of our patient ***ST3470***, experiencing primary resistance to lirafugratinib despite clearance of three *FGFR2* kinase domain mutations (Figure 5B). Considering their better on-target activity, it is possible that progression to irreversible FGFR inhibitors occurs without detectable *FGFR2* kinase domain mutations or off-target alterations, suggesting the implication of additional mechanisms, as indicated by the resistance study to futibatinib in our cohort (Figure 3C).

Given the unpredictability of resistance mechanisms and the corresponding activity of irreversible inhibitors administered in a sequential way, their administration as first anti-FGFR agents seems appropriate, in particular considering the outcomes of clinical activity reported in clinical trials in this setting (9,10). As showed here in two cases (Figure 5), switching from an irreversible inhibitor to another can also be a suitable therapeutic option.

This study, however, is not without limitations. Primarily, it relies on genomic analyses, potentially overlooking non-genetic factors like epithelial-mesenchymal transition or activation of alternate resistance pathways, as recently reported for EGFR in *FGFR2*-driven cholangiocarcinoma (41,42). Moreover, our focus is mainly on on-target resistance mechanisms, with less emphasis on proving the role of off-target events such as MAPK and PI3K/mTOR alterations. The lack of systematic tissue biopsy and ctDNA analysis at multiple timepoints for all patients may also have constrained the depth of our insights into resistance mechanisms. Finally, the lack of clinical data of resistance to lirafugratinib limits our observations of resistance to irreversible FGFR inhibitors.

In summary, the present work provides a global approach to apprehend resistance mechanisms to FGFR inhibitors across *FGFR2*-driven diseases, a clinical entity of major current interest given the development of active targeted agents. Our clinical and molecular findings were corroborated by functional analyses of *FGFR2* kinase domain mutations in conferring resistance to different FGFR inhibitors. The additional clinical experience with sequential treatment with FGFR inhibitors or everolimus, together with the concomitant longitudinal study on resistance mechanisms, provided further valuable information both on the potential clinical management of patients and on the molecular correlates of resistance in this setting.

## Supplementary Material

Supplementary Figure S1

Supplementary Figure S2

Supplementary Figure S3

Supplementary Figure S4

Supplementary Figure S5

Supplementary Table S1

Supplementary Table S2

Supplementary Table S3

Supplementary Table S4

## Data Availability

WES/RNA-seq raw data files from this study are deposited at the European Genome–phenome Archive (EGA) using the accession code EGAD50000000439. Access to this shared dataset is controlled by the institutional Data Access Committee, and requests for access can be sent to the corresponding author. Further information about EGA can be found at https://ega-archive.org/. Any additional information required to reanalyze the data reported in this article is available upon request from the corresponding author.

## Statement of Translational Relevance

The clinical benefit generated by selective FGFR inhibitors in patients with *FGFR2*-driven cancer is hampered by the inevitable occurrence of resistance. We analyzed post-progression ctDNA and tissue biopsies from 36 patients suffering from *FGFR2*-driven cholangiocarcinoma or other tumor types, at resistance to FGFR inhibitors. We were therefore able to recognize molecular traits, characteristics of resistance to reversible inhibitors *versus* the irreversible agent futibatinib, especially in terms of variety of *FGFR2* kinase domain mutations involved in resistance. Compared to *FGFR2*-driven cholangiocarcinomas, polyclonal *FGFR2* kinase domain mutations were less frequent in other tumor types. Twelve patients were treated with an additional FGFR irreversible inhibitor (futibatinib or lirafugratinib), and two derived benefit from everolimus. We integrated longitudinal molecular data with the clinical outcomes on these sequential targeted treatments, with functional evidence using Ba/F3 cellular models and patient-derived xenografts, aiming to propose molecular treatment strategies to overcome resistance.
