# Supplementary material for: Understanding and Overcoming Resistance to Selective FGFR inhibitors Across FGFR2-Driven Malignancies
Source: Clin Cancer Res. Author manuscript; Available in PMC 2024 Sep 20. (PMC7616615; doi:10.1158/1078-0432.CCR-24-1834)
Supplement: Supplementary Figure S1 [file EMS198549-supplement-Supplementary_Figure_S1.pptx]

## Slide 1
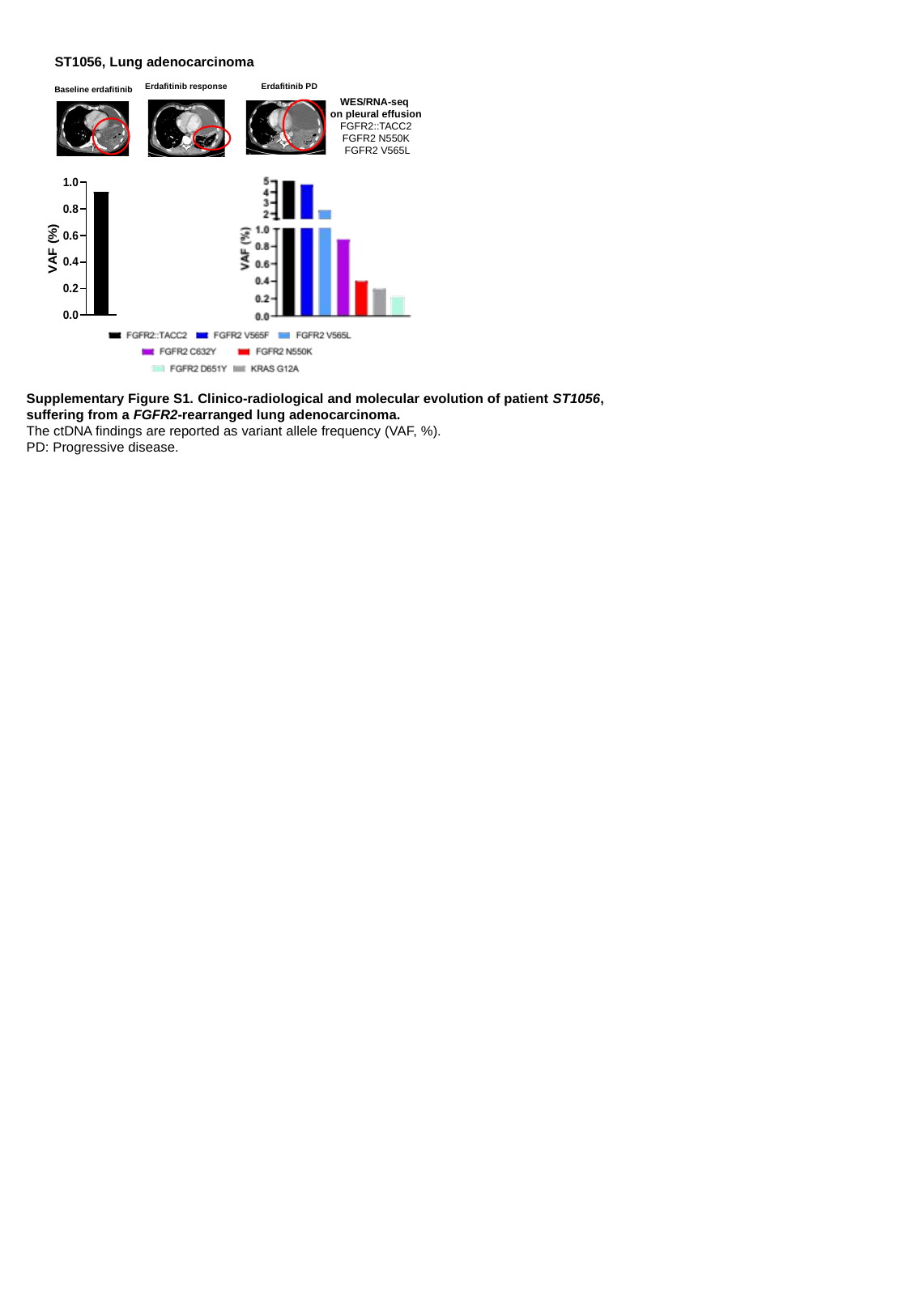

ST1056, Lung adenocarcinoma
Erdafitinib PD
Erdafitinib response
Baseline erdafitinib
WES/RNA-seq
on pleural effusion
FGFR2::TACC2
FGFR2 N550K
 FGFR2 V565L
Supplementary Figure S1. Clinico-radiological and molecular evolution of patient ST1056,
suffering from a FGFR2-rearranged lung adenocarcinoma.
The ctDNA findings are reported as variant allele frequency (VAF, %).
PD: Progressive disease.
