# Supplementary material for: Understanding and Overcoming Resistance to Selective FGFR inhibitors Across FGFR2-Driven Malignancies
Source: Clin Cancer Res. Author manuscript; Available in PMC 2024 Sep 20. (PMC7616615; doi:10.1158/1078-0432.CCR-24-1834)
Supplement: Supplementary Figure S2 [file EMS198549-supplement-Supplementary_Figure_S2.pptx]

## Slide 1
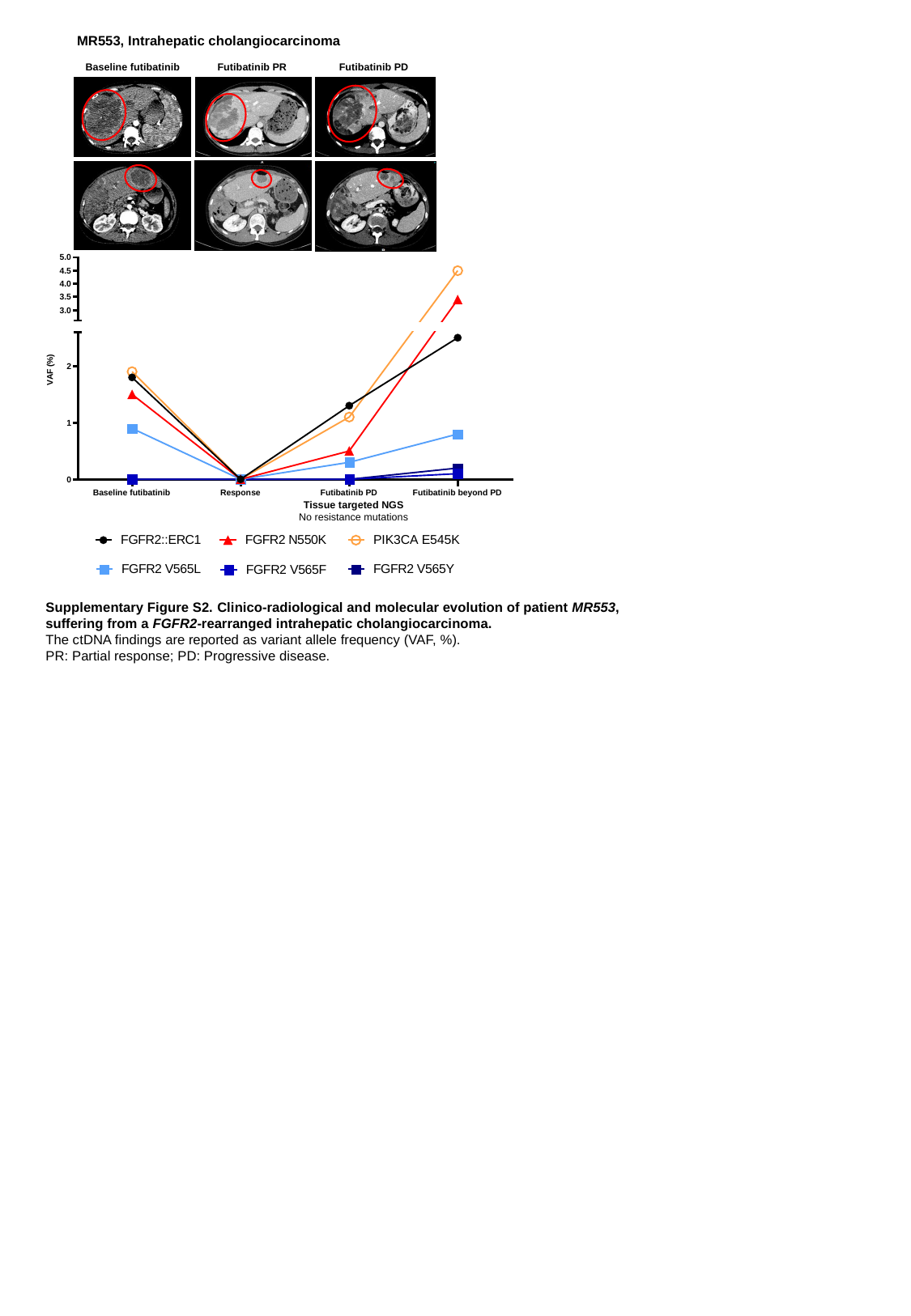

MR553, Intrahepatic cholangiocarcinoma
Baseline futibatinib
Futibatinib PR
Futibatinib PD
Tissue targeted NGS
No resistance mutations
Supplementary Figure S2. Clinico-radiological and molecular evolution of patient MR553,
suffering from a FGFR2-rearranged intrahepatic cholangiocarcinoma.
The ctDNA findings are reported as variant allele frequency (VAF, %).
PR: Partial response; PD: Progressive disease.
