# Supplementary material for: Understanding and Overcoming Resistance to Selective FGFR inhibitors Across FGFR2-Driven Malignancies
Source: Clin Cancer Res. Author manuscript; Available in PMC 2024 Sep 20. (PMC7616615; doi:10.1158/1078-0432.CCR-24-1834)
Supplement: Supplementary Figure S3 [file EMS198549-supplement-Supplementary_Figure_S3.pptx]

## Slide 1
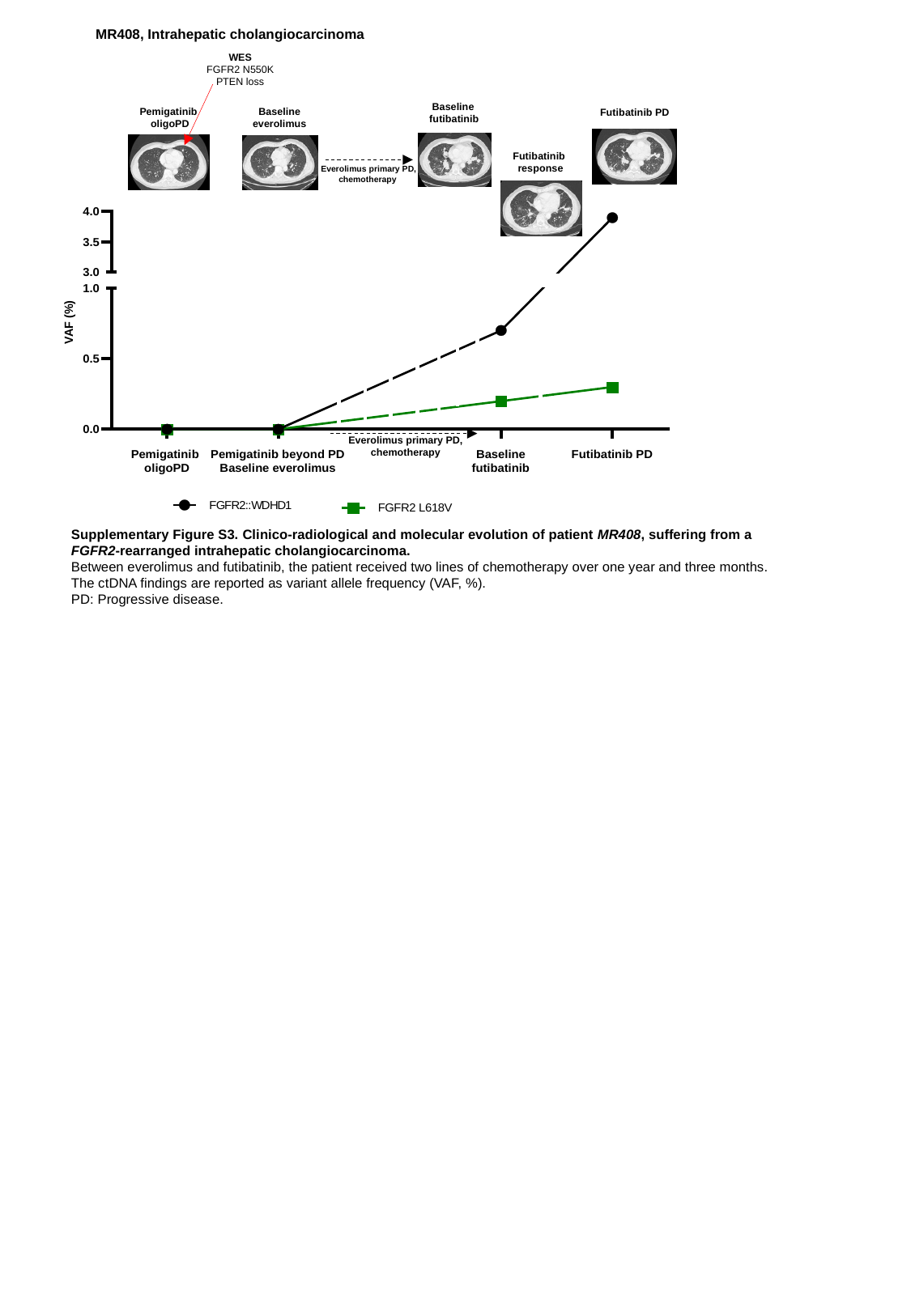

MR408, Intrahepatic cholangiocarcinoma
WES
FGFR2 N550K
PTEN loss
 Baseline
futibatinib
Pemigatinib
oligoPD
 Baseline
everolimus
Futibatinib PD
Futibatinib
response
Everolimus primary PD,
chemotherapy
Everolimus primary PD,
chemotherapy
Supplementary Figure S3. Clinico-radiological and molecular evolution of patient MR408, suffering from a FGFR2-rearranged intrahepatic cholangiocarcinoma.
Between everolimus and futibatinib, the patient received two lines of chemotherapy over one year and three months.
The ctDNA findings are reported as variant allele frequency (VAF, %).
PD: Progressive disease.
