# Supplementary material for: Understanding and Overcoming Resistance to Selective FGFR inhibitors Across FGFR2-Driven Malignancies
Source: Clin Cancer Res. Author manuscript; Available in PMC 2024 Sep 20. (PMC7616615; doi:10.1158/1078-0432.CCR-24-1834)
Supplement: Supplementary Figure S4 [file EMS198549-supplement-Supplementary_Figure_S4.pptx]

## Slide 1
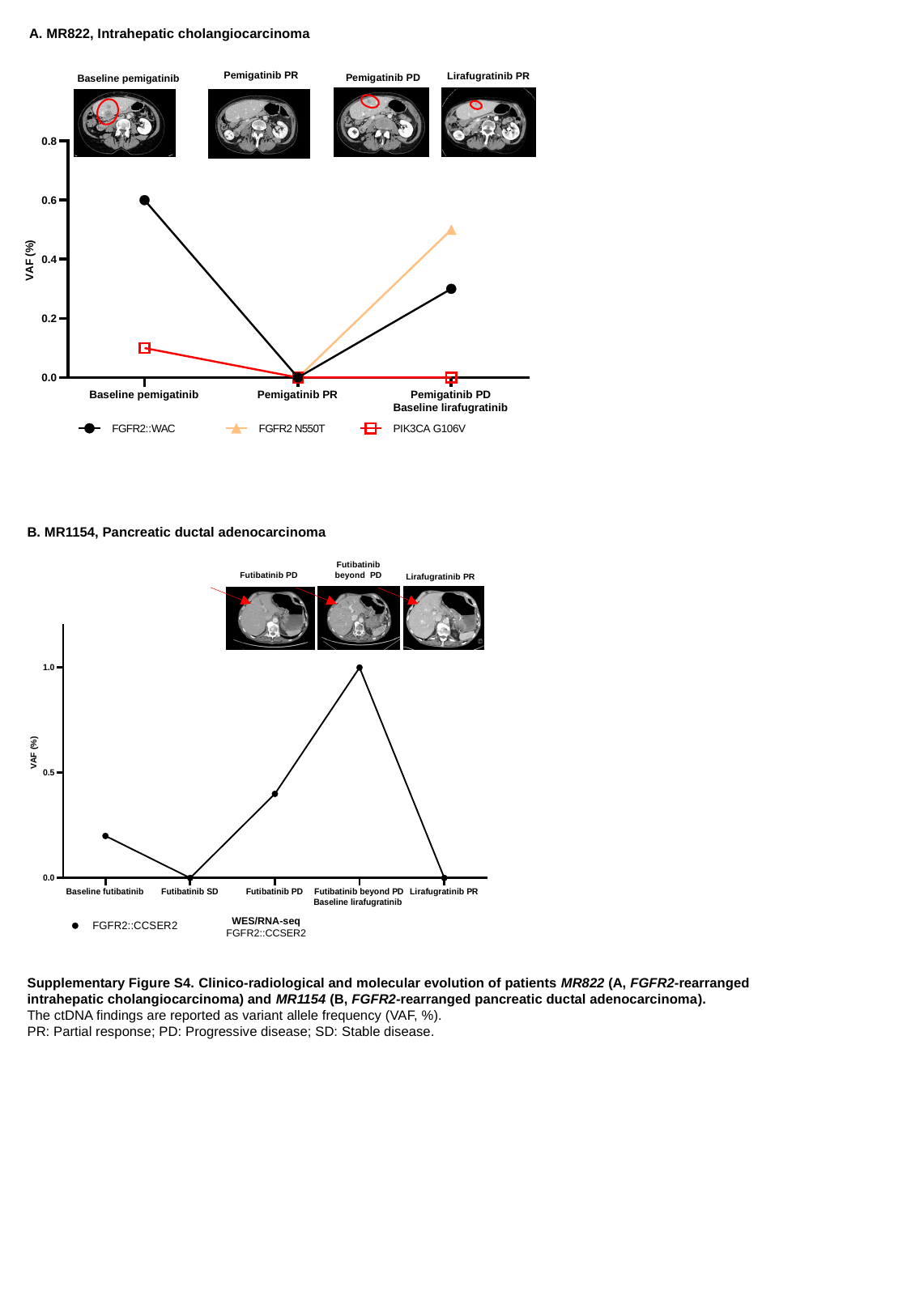

A. MR822, Intrahepatic cholangiocarcinoma
 Pemigatinib PR
 Lirafugratinib PR
 Pemigatinib PD
Baseline pemigatinib
B. MR1154, Pancreatic ductal adenocarcinoma
Futibatinib
beyond PD
Futibatinib PD
Lirafugratinib PR
WES/RNA-seq
FGFR2::CCSER2
Supplementary Figure S4. Clinico-radiological and molecular evolution of patients MR822 (A, FGFR2-rearranged intrahepatic cholangiocarcinoma) and MR1154 (B, FGFR2-rearranged pancreatic ductal adenocarcinoma).
The ctDNA findings are reported as variant allele frequency (VAF, %).
PR: Partial response; PD: Progressive disease; SD: Stable disease.
