# Supplementary material for: Understanding and Overcoming Resistance to Selective FGFR inhibitors Across FGFR2-Driven Malignancies
Source: Clin Cancer Res. Author manuscript; Available in PMC 2024 Sep 20. (PMC7616615; doi:10.1158/1078-0432.CCR-24-1834)
Supplement: Supplementary Figure S5 [file EMS198549-supplement-Supplementary_Figure_S5.pptx]

## Slide 1
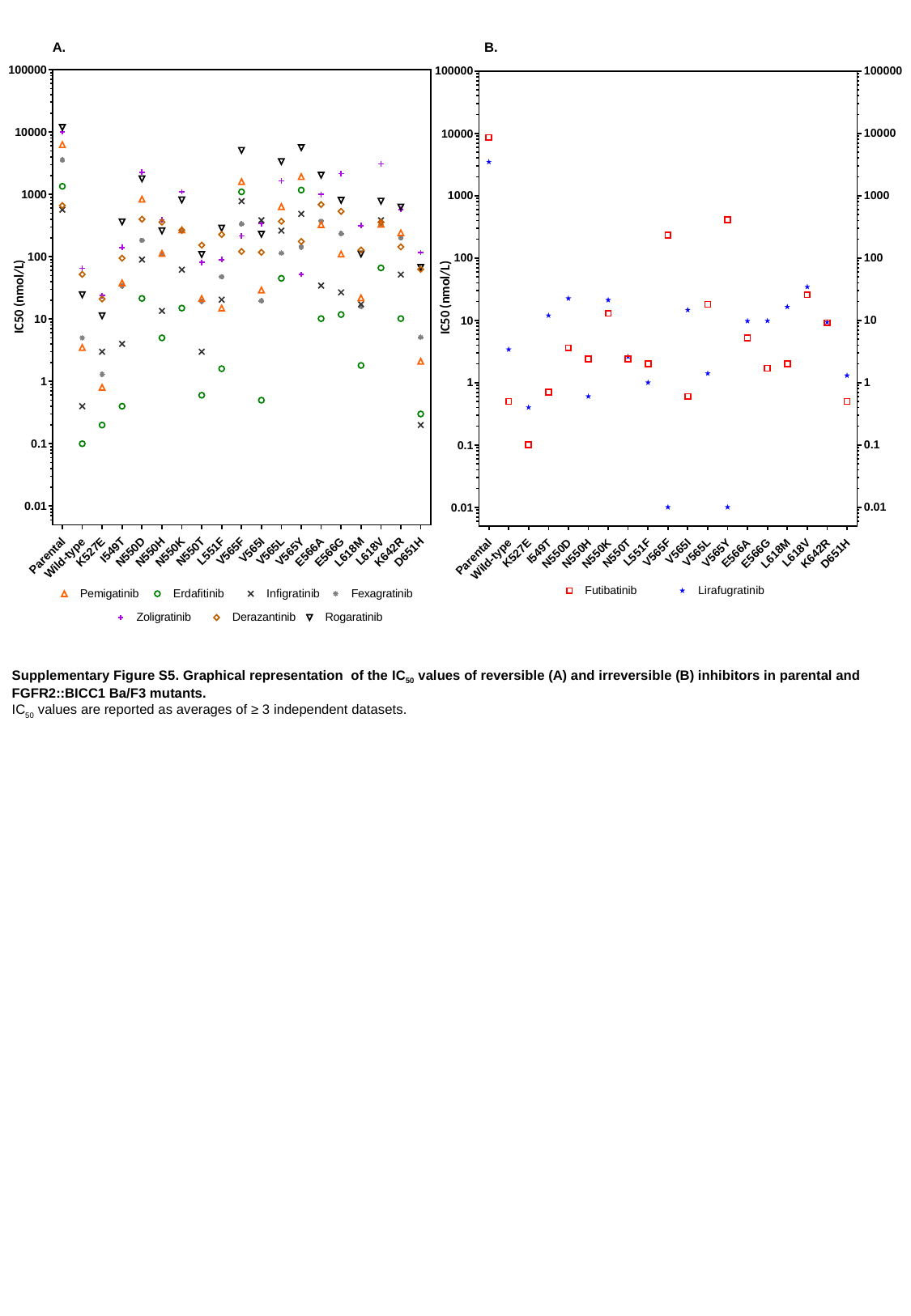

A.
B.
Supplementary Figure S5. Graphical representation of the IC50 values of reversible (A) and irreversible (B) inhibitors in parental and FGFR2::BICC1 Ba/F3 mutants.
IC50 values are reported as averages of ≥ 3 independent datasets.
