# Supplementary material for: Understanding and Overcoming Resistance to Selective FGFR inhibitors Across FGFR2-Driven Malignancies
Source: Clin Cancer Res. Author manuscript; Available in PMC 2024 Sep 20. (PMC7616615; doi:10.1158/1078-0432.CCR-24-1834)
Supplement: Supplementary Table S1 [file EMS198549-supplement-Supplementary_Table_S1.pptx]

## Slide 1
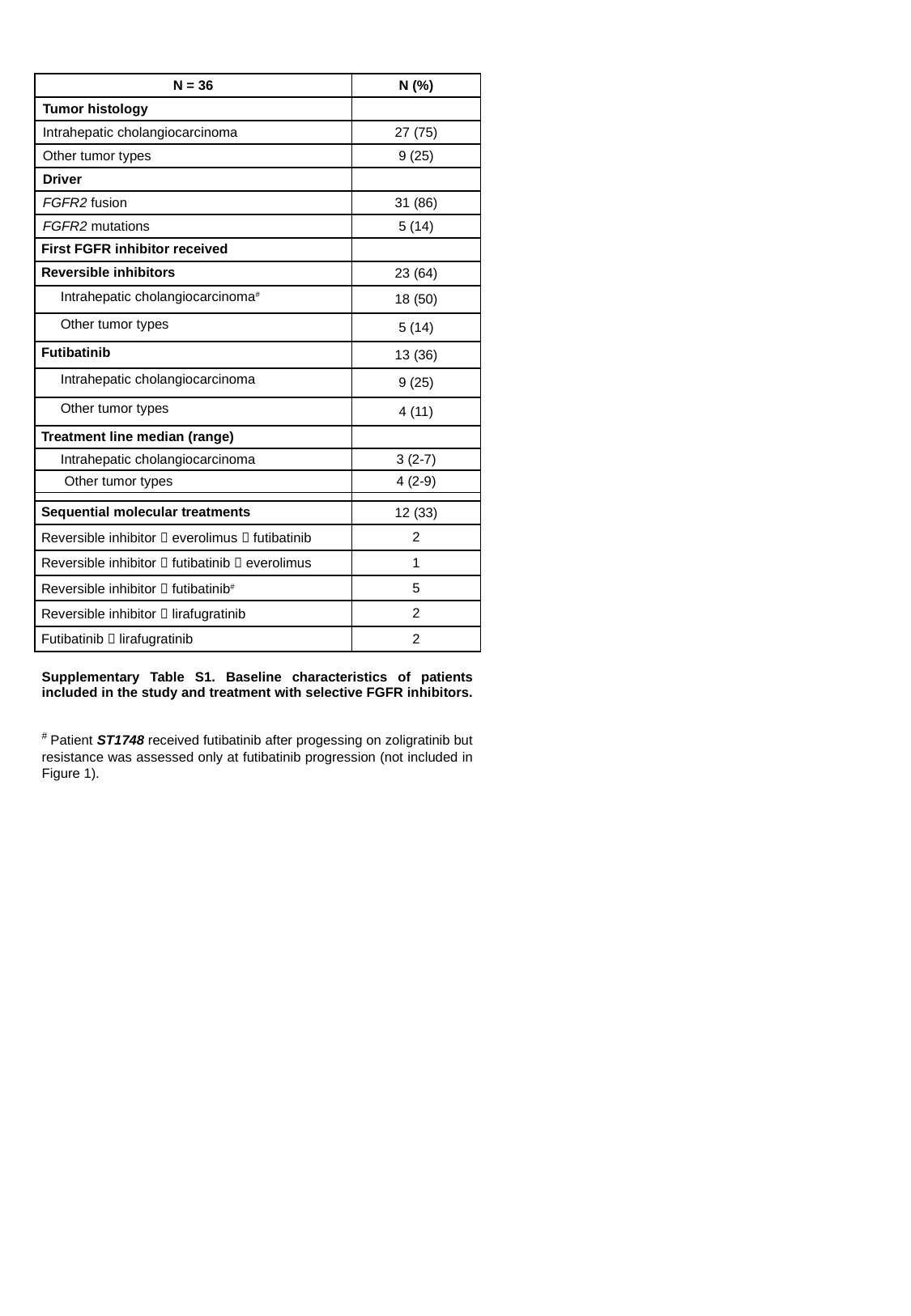

| N = 36 | N (%) |
| --- | --- |
| Tumor histology | |
| Intrahepatic cholangiocarcinoma | 27 (75) |
| Other tumor types | 9 (25) |
| Driver | |
| FGFR2 fusion | 31 (86) |
| FGFR2 mutations | 5 (14) |
| First FGFR inhibitor received | |
| Reversible inhibitors | 23 (64) |
| Intrahepatic cholangiocarcinoma# | 18 (50) |
| Other tumor types | 5 (14) |
| Futibatinib | 13 (36) |
| Intrahepatic cholangiocarcinoma | 9 (25) |
| Other tumor types | 4 (11) |
| Treatment line median (range) | |
| Intrahepatic cholangiocarcinoma | 3 (2-7) |
| Other tumor types | 4 (2-9) |
| | |
| Sequential molecular treatments | 12 (33) |
| Reversible inhibitor  everolimus  futibatinib | 2 |
| Reversible inhibitor  futibatinib  everolimus | 1 |
| Reversible inhibitor  futibatinib# | 5 |
| Reversible inhibitor  lirafugratinib | 2 |
| Futibatinib  lirafugratinib | 2 |
Supplementary Table S1. Baseline characteristics of patients included in the study and treatment with selective FGFR inhibitors.
# Patient ST1748 received futibatinib after progessing on zoligratinib but resistance was assessed only at futibatinib progression (not included in Figure 1).
