# Supplementary material for: Understanding and Overcoming Resistance to Selective FGFR inhibitors Across FGFR2-Driven Malignancies
Source: Clin Cancer Res. Author manuscript; Available in PMC 2024 Sep 20. (PMC7616615; doi:10.1158/1078-0432.CCR-24-1834)
Supplement: Supplementary Table S2 [file EMS198549-supplement-Supplementary_Table_S2.pptx]

## Slide 1
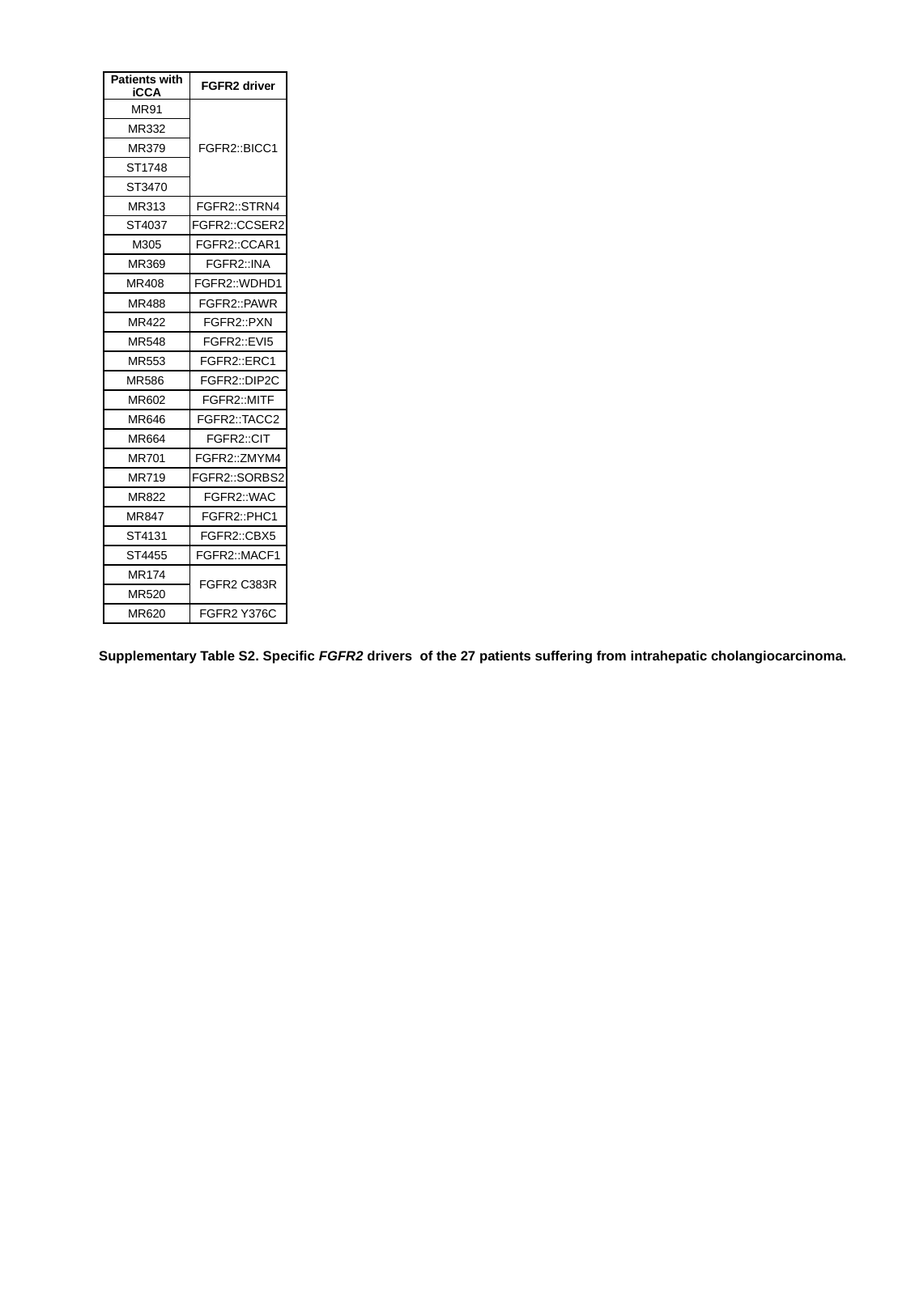

| Patients with iCCA | FGFR2 driver |
| --- | --- |
| MR91 | FGFR2::BICC1 |
| MR332 | FGFR2::BICC1 |
| MR379 | FGFR2::BICC1 |
| ST1748 | FGFR2::BICC1 |
| ST3470 | FGFR2::BICC1 |
| MR313 | FGFR2::STRN4 |
| ST4037 | FGFR2::CCSER2 |
| M305 | FGFR2::CCAR1 |
| MR369 | FGFR2::INA |
| MR408 | FGFR2::WDHD1 |
| MR488 | FGFR2::PAWR |
| MR422 | FGFR2::PXN |
| MR548 | FGFR2::EVI5 |
| MR553 | FGFR2::ERC1 |
| MR586 | FGFR2::DIP2C |
| MR602 | FGFR2::MITF |
| MR646 | FGFR2::TACC2 |
| MR664 | FGFR2::CIT |
| MR701 | FGFR2::ZMYM4 |
| MR719 | FGFR2::SORBS2 |
| MR822 | FGFR2::WAC |
| MR847 | FGFR2::PHC1 |
| ST4131 | FGFR2::CBX5 |
| ST4455 | FGFR2::MACF1 |
| MR174 | FGFR2 C383R |
| MR520 | FGFR2 C383R |
| MR620 | FGFR2 Y376C |
Supplementary Table S2. Specific FGFR2 drivers of the 27 patients suffering from intrahepatic cholangiocarcinoma.
