# Supplementary material for: Understanding and Overcoming Resistance to Selective FGFR inhibitors Across FGFR2-Driven Malignancies
Source: Clin Cancer Res. Author manuscript; Available in PMC 2024 Sep 20. (PMC7616615; doi:10.1158/1078-0432.CCR-24-1834)
Supplement: Supplementary Table S3 [file EMS198549-supplement-Supplementary_Table_S3.pptx]

## Slide 1
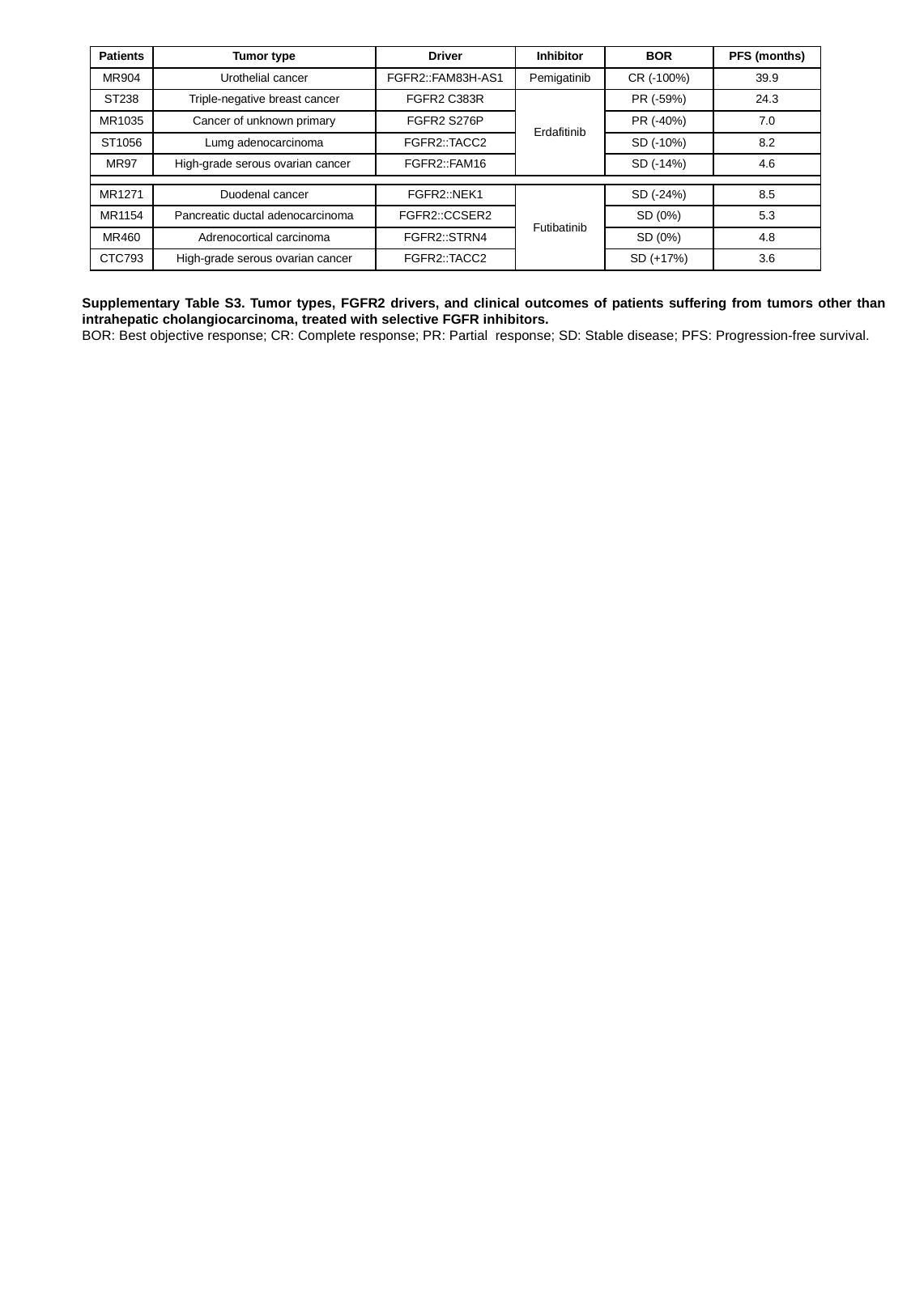

| Patients | Tumor type | Driver | Inhibitor | BOR | PFS (months) |
| --- | --- | --- | --- | --- | --- |
| MR904 | Urothelial cancer | FGFR2::FAM83H-AS1 | Pemigatinib | CR (-100%) | 39.9 |
| ST238 | Triple-negative breast cancer | FGFR2 C383R | Erdafitinib | PR (-59%) | 24.3 |
| MR1035 | Cancer of unknown primary | FGFR2 S276P | Erdafitinib | PR (-40%) | 7.0 |
| ST1056 | Lumg adenocarcinoma | FGFR2::TACC2 | Erdafitinib | SD (-10%) | 8.2 |
| MR97 | High-grade serous ovarian cancer | FGFR2::FAM16 | Erdafitinib | SD (-14%) | 4.6 |
| | | | | | |
| MR1271 | Duodenal cancer | FGFR2::NEK1 | Futibatinib | SD (-24%) | 8.5 |
| MR1154 | Pancreatic ductal adenocarcinoma | FGFR2::CCSER2 | Futibatinib | SD (0%) | 5.3 |
| MR460 | Adrenocortical carcinoma | FGFR2::STRN4 | Futibatinib | SD (0%) | 4.8 |
| CTC793 | High-grade serous ovarian cancer | FGFR2::TACC2 | Futibatinib | SD (+17%) | 3.6 |
Supplementary Table S3. Tumor types, FGFR2 drivers, and clinical outcomes of patients suffering from tumors other than intrahepatic cholangiocarcinoma, treated with selective FGFR inhibitors.
BOR: Best objective response; CR: Complete response; PR: Partial response; SD: Stable disease; PFS: Progression-free survival.
