# Supplementary material for: Understanding and Overcoming Resistance to Selective FGFR inhibitors Across FGFR2-Driven Malignancies
Source: Clin Cancer Res. Author manuscript; Available in PMC 2024 Sep 20. (PMC7616615; doi:10.1158/1078-0432.CCR-24-1834)
Supplement: Supplementary Table S4 [file EMS198549-supplement-Supplementary_Table_S4.pptx]

## Slide 1
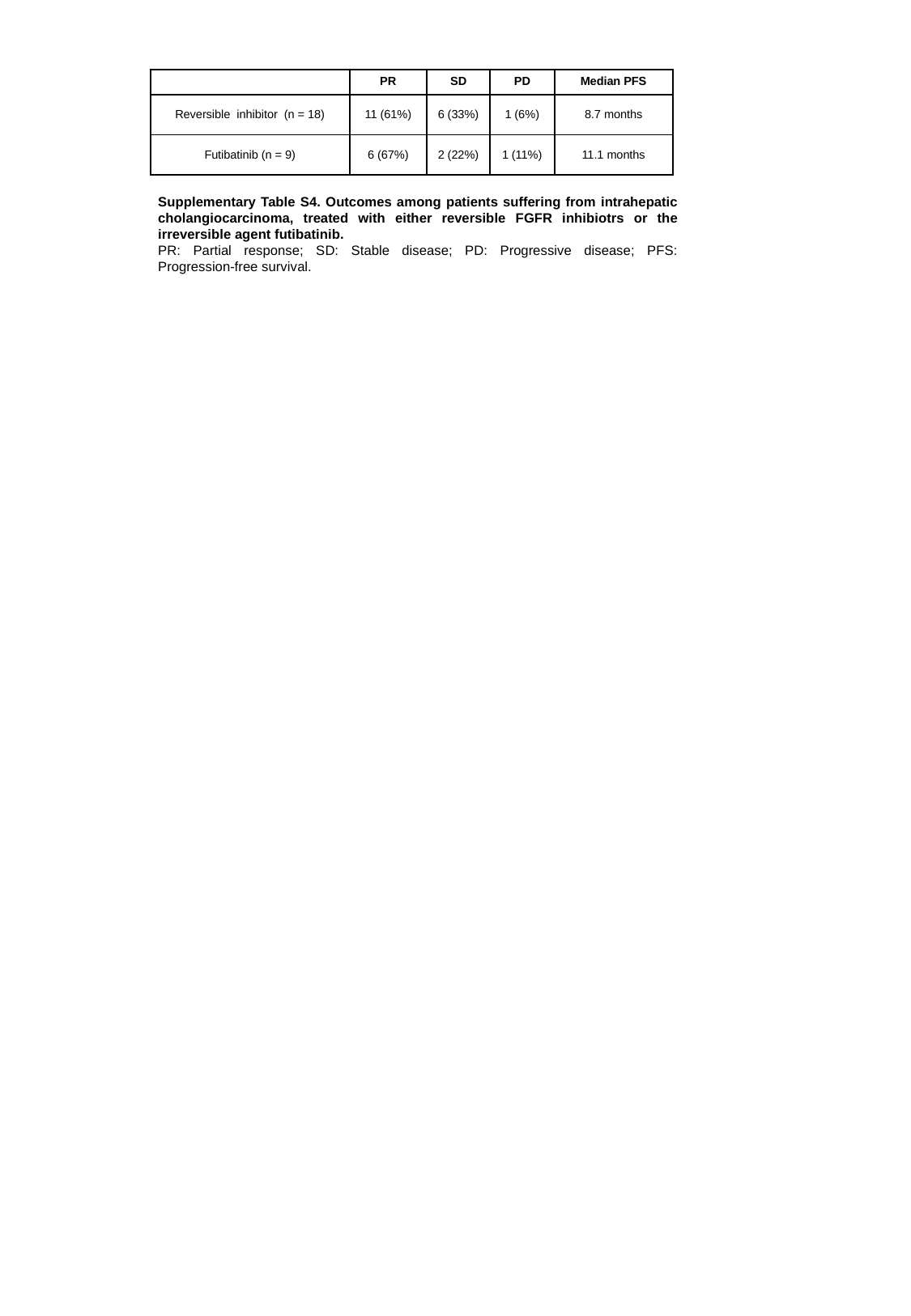

| | PR | SD | PD | Median PFS |
| --- | --- | --- | --- | --- |
| Reversible inhibitor (n = 18) | 11 (61%) | 6 (33%) | 1 (6%) | 8.7 months |
| Futibatinib (n = 9) | 6 (67%) | 2 (22%) | 1 (11%) | 11.1 months |
Supplementary Table S4. Outcomes among patients suffering from intrahepatic cholangiocarcinoma, treated with either reversible FGFR inhibiotrs or the irreversible agent futibatinib.
PR: Partial response; SD: Stable disease; PD: Progressive disease; PFS: Progression-free survival.
